# Size-exclusion chromatography as a multi-attribute method for process and product characterization of adeno-associated virus

**DOI:** 10.1016/j.omtm.2024.101382

**Published:** 2024-11-19

**Authors:** Sri Hari Raju Mulagapati, Arun Parupudi, Tomasz Witkos, Nick Bond, Xiaoyu Chen, Thomas Linke, Guoling Xi, Albert Ethan Schmelzer, Wei Xu

**Affiliations:** 1 Process and Analytical Sciences, BioPharmaceuticals Development (BPD), R&D, AstraZeneca, Gaithersburg, MD, USA; 2 Viral Vector Product Development, Process Development and Clinical Supply, Alexion, Boston, MA, USA; 3 Drug Product and Formulation Sciences, GSK Vaccines, Rockville, MD 20850, USA

**Keywords:** size-exclusion chromatography, multi-attribute monitoring, MAM, adeno-associated viruses, different in-line detectors, product quality attributes, quality control method, high-throughput method, SEC-HPLC, AAV

## Abstract

Adeno-associated viruses (AAVs) have recently emerged as a leading platform for gene therapy. Due to the complex manufacturing process and structural features of AAVs, extensive process and product characterization studies are required to better understand product quality and batch-to-batch variability. It is, therefore, critical to develop a fast and reliable analytical method to monitor different product quality attributes (PQAs) of AAVs. In this study, we developed a multiple-attribute monitoring (MAM) method for the characterization of AAV PQAs. The MAM method was developed using the separation capability of size-exclusion chromatography (SEC) in connection with multiple in-line detectors: ultraviolet (UV), fluorescence (FLD), multi-angle light scattering (MALS), and refractive index (RI). We demonstrate that our SEC-based MAM method can be used to measure different PQAs, including genome and capsid titer, purity, aggregation, and full/empty capsid ratios in a single assay. Our SEC-based MAM method achieves similar results when compared side by side with orthogonal, individual assays such as quantitative polymerase chain reaction (qPCR), enzyme-linked immunosorbent assay (ELISA), and anion-exchange chromatography (AEX). Moreover, here we demonstrate that a simple, label-free, cost-effective, minimum sample requirement, and a high-throughput method can be applied to support process development, product characterization, release, and stability testing.

## Graphical abstract

**Figure:**
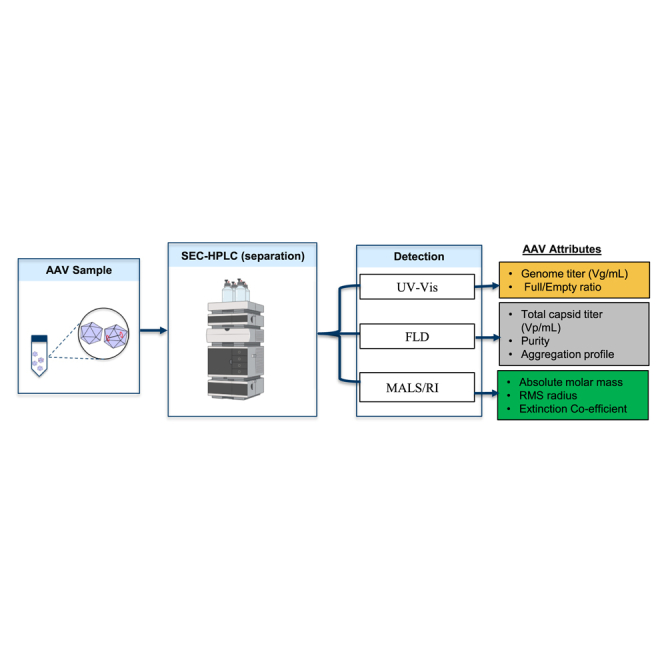

## Teaser

Mulagapati and colleagues use size-exclusion chromatography with in-line detectors (UV, FLD, MALS, RI) to simultaneously evaluate quality attributes of adeno-associated viruses, including genome and capsid titer, purity, aggregation, and full/empty capsid ratios for product and process characterization.

## Introduction

Adeno-associated virus (AAV) has emerged as a leading platform for *in vivo* gene therapy due to its safety profile, long-term expression ability, and efficient transduction of various target tissues.^1^ Ongoing clinical trials and recent regulatory approvals (including LUXTURNA [voretigene neparvovec-rzyl], ZOLGENSMA [onasemnogene abeparvovec-xioi], ROCTAVIAN [valoctocogene roxaparvovec-rvox], and HEMGENIX [etranacogene dezaparvovec-drlb]) have shown AAV-based gene therapy as a breakthrough milestone to treat a wide range of diseases.^2^ AAVs are single-stranded DNA (ssDNA), non-enveloped viruses that belong to the Parvoviridae family and Dependoparvovirus genus. AAV particles are composed of viral proteins (60 monomers) and ssDNA (∼4.7 kb), which interact to form a capsid particle (at ∼25 nm size) arranged into an icosahedral structure. The 60 viral proteins are a mixture of three types of structural proteins (VP1, 2, and 3) in a ratio of 1:1:10.^3^^,^^4^

AAVs are referred to as recombinant AAV (rAAV) when used as a viral vector to deliver therapeutic transgenes. rAAV production mostly relies on processes that can ensure a high-quality product, are scalable, and achieve cost-effective manufacturing.^5^ Overall, the large-scale production of rAAV depends on several variables, including (1) optimization of upstream including expression of capsid proteins (VP1, 2, and 3), transgene, and proteins that facilitate assembly; (2) downstream, including efficient cell lysis, clarification, and chromatography steps to achieve target product quality specifications; and (3) formulation development.^6^ It is essential to closely monitor product quality, maintain batch-to-batch consistency, and control any undesired process- and product-related impurities during production and storage of rAAVs.^7^

Successful rAAV production requires a strong analytical platform that can monitor product quality, in-process analysis, product stability, and batch-to-batch variability.^7^ In-depth analytical characterization is more complex for AAVs than for traditional well-characterized biologics (e.g., monoclonal antibodies [mAbs], recombinant proteins) because AAVs are composed of large viral capsid proteins (VP1, VP2, and VP3) that encapsulate DNA, which together makes the analytical characterization challenging.^8^ In recent years, a variety of quality control (QC) release and extended characterization methods were developed to control potency, identity, purity, and product- and process-related impurities.^9^ The most common analytical methods, such as enzyme-linked immunosorbent assay (ELISA), quantitative polymerase chain reaction (qPCR), analytical ultracentrifugation (AUC), electron microscopy (EM), UV-visible (UV-vis) spectroscopy, high-performance liquid chromatography (HPLC), and liquid chromatography-mass spectrometry (LC-MS), were applied independently to monitor different AAV product quality attributes (PQAs) such as genome and capsid titer, purity, aggregation, morphology, full/empty (F/E) capsid ratio, capsid identities, and post-translation modifications (PTMs).^4^^,^^7^^,^^10^^,^^11^^,^^12^^,^^13^^,^^14^^,^^15^ These methods are useful for characterizing rAAV; however, each of the methods listed above has some limitations, such as low throughput, high material demand, need for highly skilled analysts, high variability, serotype specificity, and sample purity.^16^ To overcome some of these limitations, it is desirable to develop an effective multiple-attribute monitoring (MAM) method to characterize different AAV PQAs within a single assay.^17^ Over the last few years, MAM methods have gained significant attention for development and production of therapeutic proteins (e.g., mAbs) as a high-throughput and cost-effective option to better understand and monitor both the manufacturing process and PQAs.^17^^,^^18^ In this study, we developed a size-exclusion chromatography (SEC) method coupled with multiple in-line detectors as an MAM approach for comprehensive characterization of rAAV PQAs. The MAM method utilized SEC combined with UV, fluorescence (FLD), multi-angle light scattering (MALS), and refractive index (RI) detectors. Each detector provided valuable information for different PQAs: SEC-UV (260- and 280-nm absorbance ratio) for full/empty capsid ratio; SEC-FLD for purity and total capsid titer (vp/mL) measurement; SEC-UV (260 nm) for genome titer (vg/mL); SEC-UV-RI-MALS for absolute molar mass (units: megadaltons [MDa] or g/mol), size (nm), and extinction coefficients (ECs or ϵ). Furthermore, we investigated the correlation between full/empty capsid ratio and SEC-UV-HPLC peak areas (at 260 and 280 nm) and ECs (at ϵ260, ϵ280). The SEC-based MAM method demonstrated comparable results to orthogonal methods for rAAV characterization. Overall, SEC-based MAM is a valuable analytical tool that is simple, label free, cost-effective, low sample demand, and a high-throughput method for extended characterization of rAAV that can be applicable for all the serotypes of rAAV in both process development and the QC environment.

## Results

### Purity measurement of rAAV by the SEC-FLD method

Our initial efforts to develop an optimized SEC separation involved evaluating multiple parameters, including different column chemistries, pore sizes, particle sizes, and mobile phase compositions. Figure S1 illustrates the chromatographic profiles from the method development studies, specifically highlighting the effect of NaCl salt concentration on AAV6.2 separation. The results indicated that a salt concentration of 300 mM provided better resolution in the separation of AAV monomers (single individual capsids) and aggregate species (double, triple, and quadruple capsids) along with good total column recovery. In contrast, a lower salt concentration of 150 mM resulted in reduced resolution and increased peak tailing, while a higher salt concentration of 500 mM led to lower recovery from the column and potential salting out of AAV, making it prone to aggregation that is method induced rather than an inherent sample property.

Additionally, Figure S2 presents chromatographic profiles demonstrating the impact of varying column chemistry, particle size, and pore size on AAV6.2 separation. In comparing the Sepax SRT columns with pore sizes of 500 and 1,000 Å, the 500-Å column provided superior resolution for AAV6.2 separation with minimal peak tailing. A 1,000-Å pore size in SEC may be too large for effective separation of AAVs (20–25 nm), as they may not sufficiently interact with the pores, leading to reduced resolution. Optimal resolution in SEC is achieved when analytes can partially penetrate the pores, facilitating size-based separation. However, with excessively large pores (1,000 Å), larger analytes like AAVs may pass through too quickly, resulting in diminished resolution. This larger pore size also risks co-elution with smaller impurities and reduces sensitivity to aggregates, impacting purity assessment.^19^ The Sepax SRT column with a 500-Å pore size and 5-μm particle size demonstrated comparable resolution in the separation of aggregates to columns with smaller particle sizes (3.5/2.5 μm) from Waters. Although smaller particle sizes typically enhance column efficiency and produce sharper peaks, they can also increase the likelihood of non-ideal interactions and higher backpressure, which can intensify tailing due to mechanical shear stress. Under these conditions, fragile AAV particles are more susceptible to degradation, including capsid disruption, aggregation, or fragmentation, thereby compromising sample integrity. Additionally, the high backpressure can limit the usability of the SEC column and complicate coupling with multiple detectors like MALS, which might add further backpressure and complicate the overall analysis. Figure S2 shows intensify tailing on the smaller particle size column compared to 5-μm particle size SRT SEC column.

Based on the resolution and column behavior in separating AAV monomer (single individual capsid) and aggregate species (double/triple/quadruple capsid), we selected an optimized condition. The optimized method was achieved using a Sepax SRT SEC-500 column with 5-μm particle size, 500-Å pore size, and dimensions of 7.8 × 300 mm. The isocratic mobile phase consisted of 20 mM sodium phosphate, 300 mM NaCl, and 10 mM KCl at pH 7.0, with a flow rate of 1.0 mL/min for a duration of 30 min.

**Figure 1:**
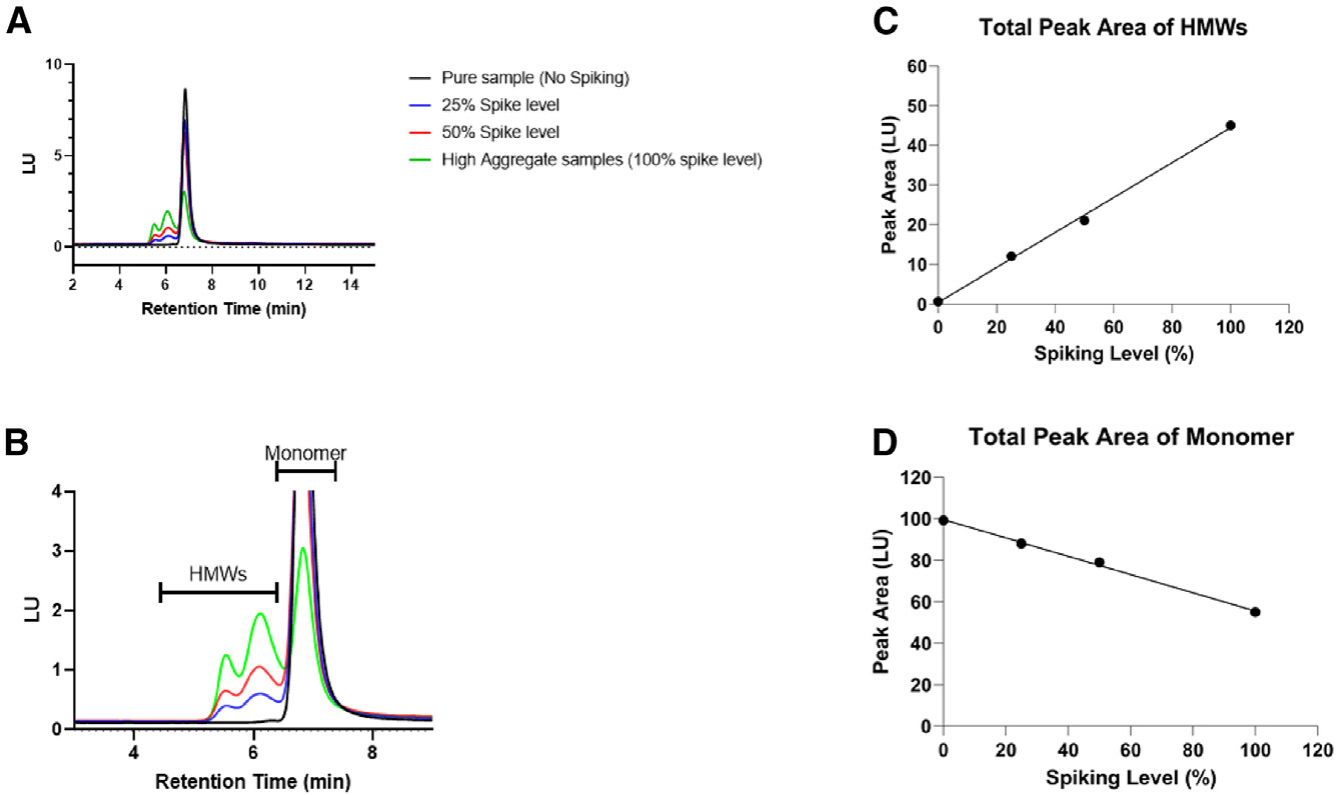
SEC-HPLC-FLD for purity measurements (A) Chromatographic profiles demonstrating rAAV purified reference (0% HMWs), high aggregated rAAV material (∼45% HMWs), and two spiked samples (high aggregated material spiked at 25% or 50% v/v in pure reference rAAV material). (B) Magnified view of the chromatographic profiles in (A), highlighting the peaks corresponding to monomer and HMWs. (C) Plot illustrating the relationship between % spiking level and fluorescence emission % peak area (HMWs) determined by SEC-FLD. (D) Plot illustrating the relationship between % spiking level and fluorescence emission % peak area (monomer) determined by SEC-FLD.

An in-house purified rAAV reference and a highly aggregated rAAV material was used for the SEC method development. Figures 1A and 1B (magnified view) show an overlay of the SEC-HPLC chromatograms of purified rAAV reference free of high-molecular-weight species (HMWs), highly aggregated material with ∼45% HMWs, and two samples spiked to different levels of full rAAV capsid. The chromatographic peaks were monitored by fluorescence (excitation = 293 nm; emission = 350 nm). The chromatographic profiles demonstrate the capability of the SEC method in resolving dimer and oligomer aggregate species of rAAV from the monomeric form. The accuracy of the method was demonstrated in a spiking study; the pure reference standard of rAAV (0% HMWs) was spiked with highly aggregated material of rAAV (containing 45% HMWs). The recovery, as a function of % total peak area of HMWs and monomer, is plotted against the % spiking level in Figures 1C and 1D, respectively. Figures 1C and 1D show good linearity (R^2^ > 0.99) and recovery (90%–110%) for rAAV monomer and HMWs with the optimized SEC-FLD method. A high level of precision was observed, with the variation not exceeding 5% relative standard deviation (RSD).

### Measurement of absolute molar mass (MDa) and root-mean-square radius (nm) of rAAV by SEC-UV-RI-MALS

**Figure 2:**
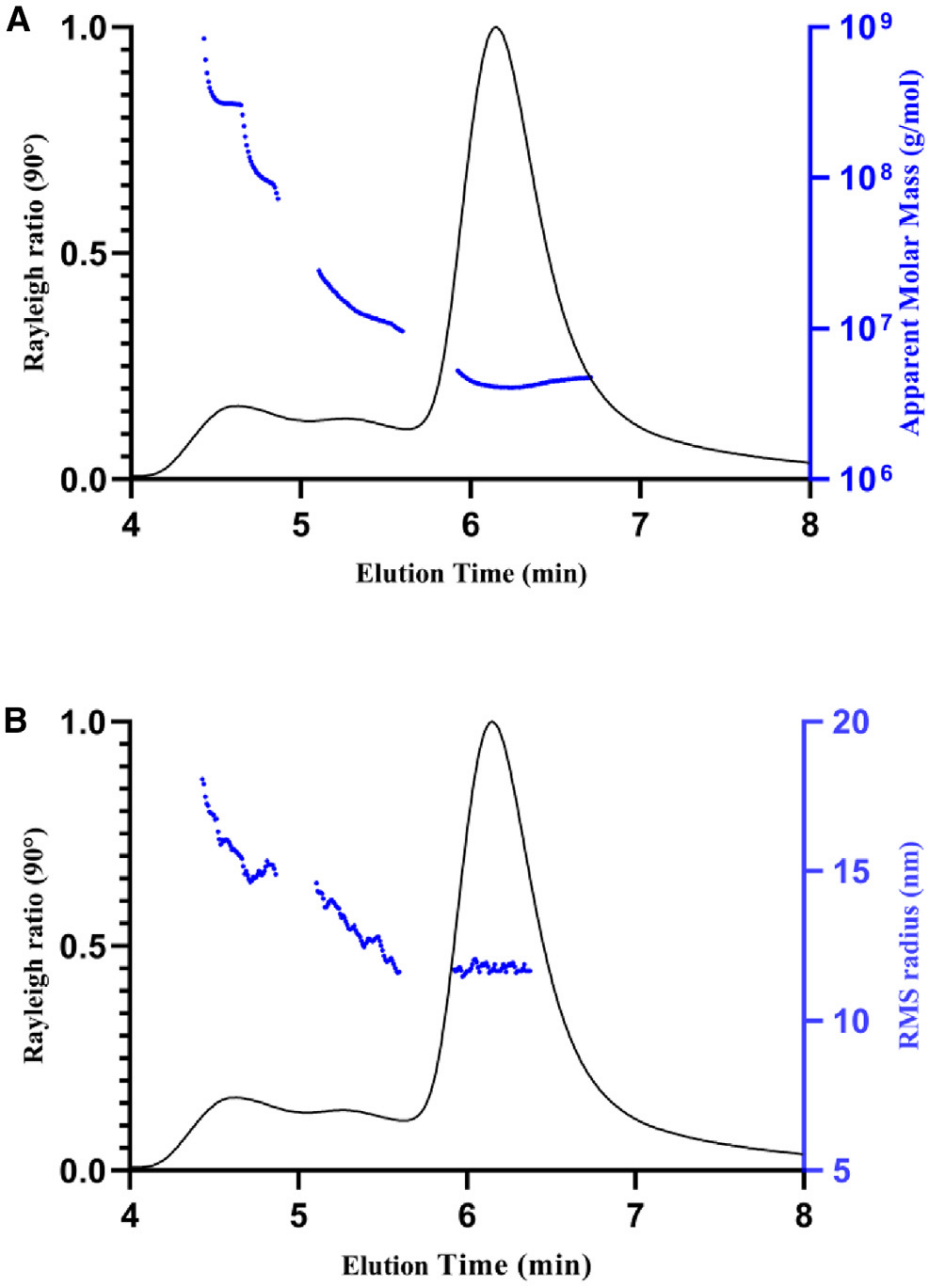
SEC-UV-RI-MALS analysis of rAAV (A) SEC-UV-MALS-RI showing light scattering signal with the absolute molar mass M_w_ (g/mol). The M_w_ of the monomer and aggregates are highlighted in blue. (B) SEC-UV-MALS-RI showing light scattering signal with RMS radius (Rg).

Figures 2A and 2B present a typical SEC-MALS chromatograms for high aggregate rAAV material showing the absolute molar mass (g/mol or MDa) and root-mean-square (RMS) radius (nm), respectively. The retention times (RTs) for the monomer and HMW peaks were approximately in the range of 6.0–7.0 min and 4.0–5.8 min, respectively. The light-scattering intensity profile (at 90° scattering angle) was overlaid with MALS-determined apparent molar mass (g/mol) in Figure 2A and RMS radius in Figure 2B, respectively. A similar pattern was observed for both parameters: the monomer peak was monodispersed (as indicated by flat molar mass and RMS profiles), while the HMW peaks had a wide range of heterogeneous molar mass and RMS values. The calculated average molar mass of the rAAV main peak was 4.2 MDa, and the calculated average RMS radius was around 11–13 nm. For HMW peaks, heterogeneous populations were observed with an average apparent molar mass range of approximately 8–100 MDa and RMS radius range of approximately 10–25 nm.

### Measurement of total capsid titer (vp/mL) and genome titer (vg/mL) of rAAV by SEC-UV (A260)-FLD method

**Figure 3:**
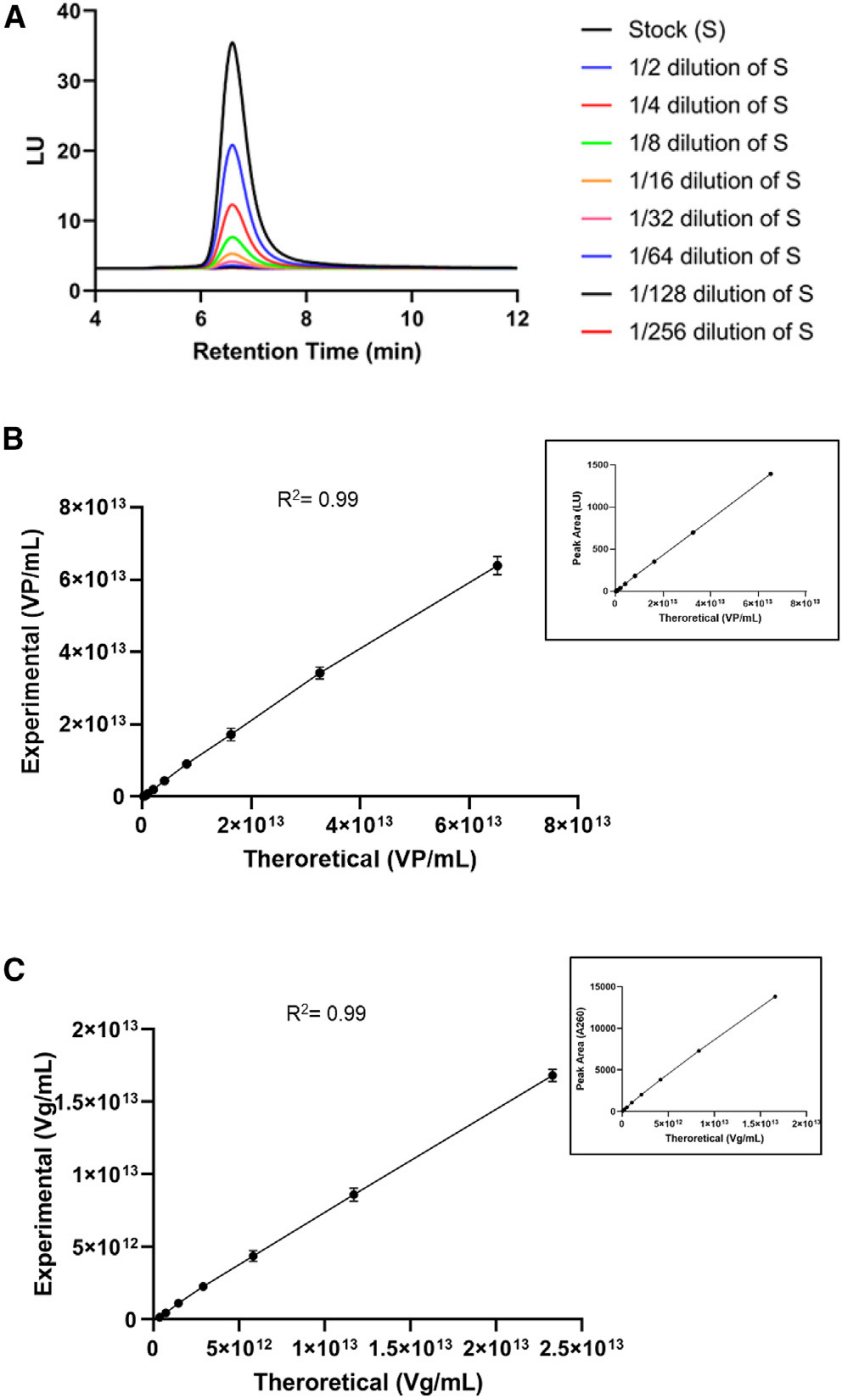
SEC-UV (A260)-FLD method for measuring total capsid (vp/mL) and genome titer (vg/mL) of rAAV (A) Overlay of SEC-FLD profiles obtained from a dilution series of rAAV samples. (B) Linearity curve illustrating the relationship between theoretical concentration (vp/mL) and the peak area of SEC-HPLC (FLD) peak. (C) Linearity curve illustrating the relationship between theoretical concentration and the peak area of SEC-HPLC (UV A260) peak, corresponding to genome titer (vg/mL).

Both total capsid titer (vp/mL) and genome titer (vg/mL) measurements of rAAV are critical parameters that need to be accurately quantified to determine the actual dose administered to the patients. Some traditional methods, such as ELISA and qPCR, are routinely used to quantify vp/mL and vg/mL titer respectively. These methods are pretty reliable but require vigorous sample preparations, well-characterized reagents, and have lower precision (∼20% CV). The SEC-UV (A260)-FLD chromatographic method evaluated here showed the capability of quantifying vp/mL and vg/mL titer in a single assay with higher precision (∼15% CV) and throughput compared to traditional methods. The SEC-FLD profiles of the dilution series standards are shown in Figures 3A. Figure 3B shows the correlation between total capsid titer (vp/mL) values against the SEC-FLD peak areas, which demonstrated good linearity (R^2^ = 0.99). A similar linear trend (R^2^ = 0.99) was also achieved for the viral genome titer (vg/mL) and SEC (UV-A260) peak areas (Figure 3C). The precision or variability of the assay, determined by analyzing three replicates of each dilution series standard, showed a % RSD ≤10 for both the total capsid titer and viral genome titer. The limit of quantitation (LOQ) and limit of detection (LOD) for the SEC-FLD method were calculated as 9.4 × 10^11^ vp/mL and 3.1 × 10^11^ vp/mL. For the SEC-UV (A260) method, the LOQ and LOD were calculated as 2.4 × 10^11^ vg/mL and 8 × 10^10^ vg/mL, respectively. Based on the results from the standard curves, SEC-FLD and SEC-UV (A260) methods demonstrate the capability of measuring multiple titers of rAAV including the total capsid titer (which includes both empty and full capsid particles) and viral genome titer (only includes full capsid particles).

**Figure 4:**
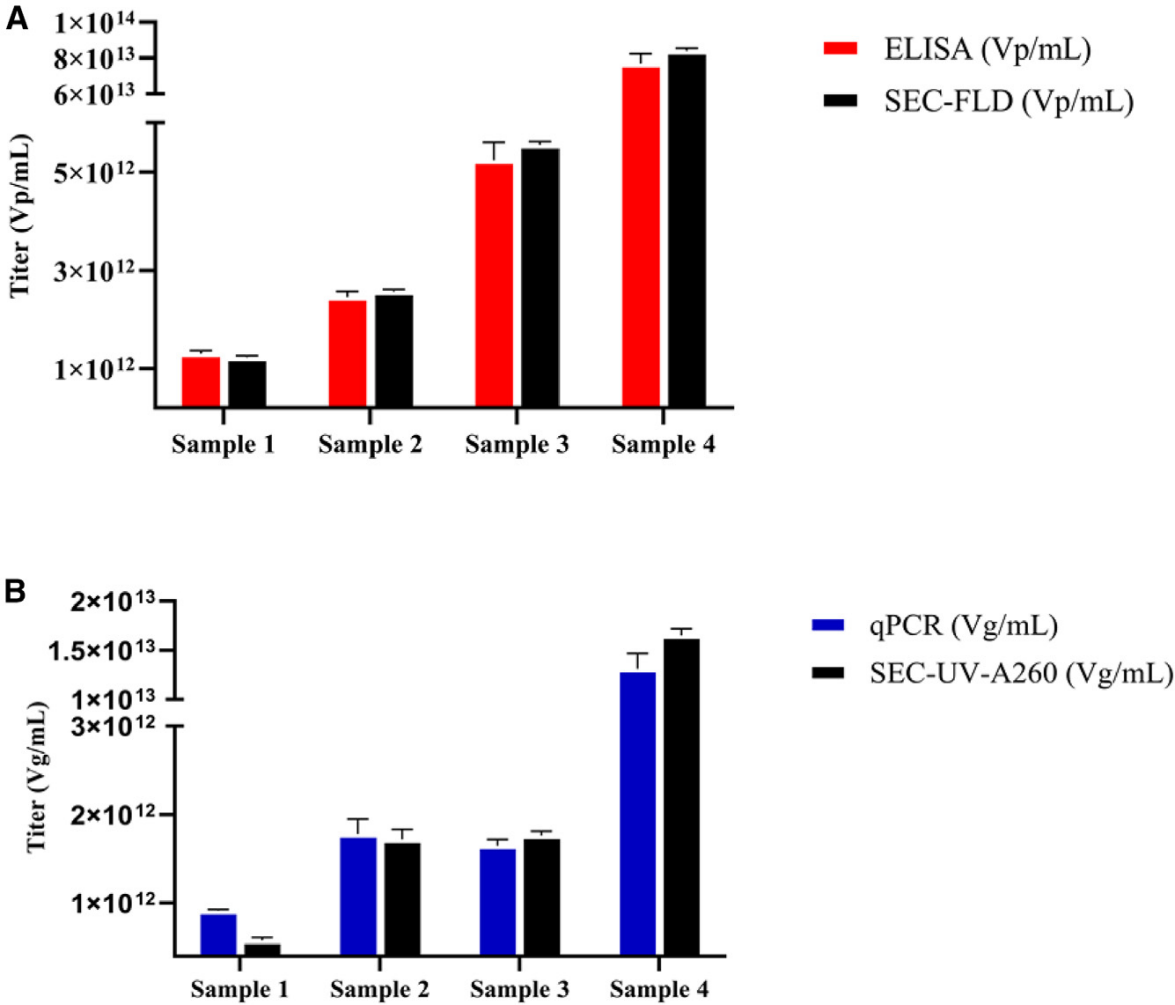
Comparative analysis of orthogonal analytical methods for qualification of total capsid (vp/mL) and genome (vg/mL) titers (A) Side-by-side comparison plot showcasing the results of ELISA and SEC-FLD measurements for total capsid titer (vp/mL). (B) Side-by-side comparison plot showcasing the results of qPCR and SEC-UV (A260) measurements for genome titer (vg/mL). The comparative analysis was conducted using in-house manufactured samples from different batches (lots).

The titer measured by the SEC-UV (A260)-FLD method was compared to the titers measured with orthogonal methods. For this comparison, four different batches (independent samples) of rAAV were used. Figure 4A shows the comparability results in vp/mL of the traditional ELISA method with the SEC-FLD method. The comparison of the results between SEC-FLD and ELISA methods demonstrates a good correlation (within 15% of difference). Figure 4B shows the comparability results of a traditional qPCR method with the SEC-UV (A260) method. The results show a good correlation (% difference is below 15%) for rAAV samples that were in the range 1 × 10^12^ to 1 × 10^13^ vg/mL. In contrast, for low-concentration rAAV samples (<1 × 10^12^ vg/mL), the % difference was higher between qPCR and SEC- UV(A260). The higher % difference between the methods at low concentrations can likely be attributed to the differences in the respective LOQs. The sensitivity of the qPCR method is better at lower concentrations, with an LOQ of 1 × 10^9^ vg/mL, when compared to the SEC- UV(A260) method, with an LOQ of 2.4 × 10^11^ vg/mL.

### Correlation of full/empty ratio of AAV with ECs (ϵ260, ϵ280) and absorbance peak areas of SEC-UV-(A260, A280)

**Figure 5:**
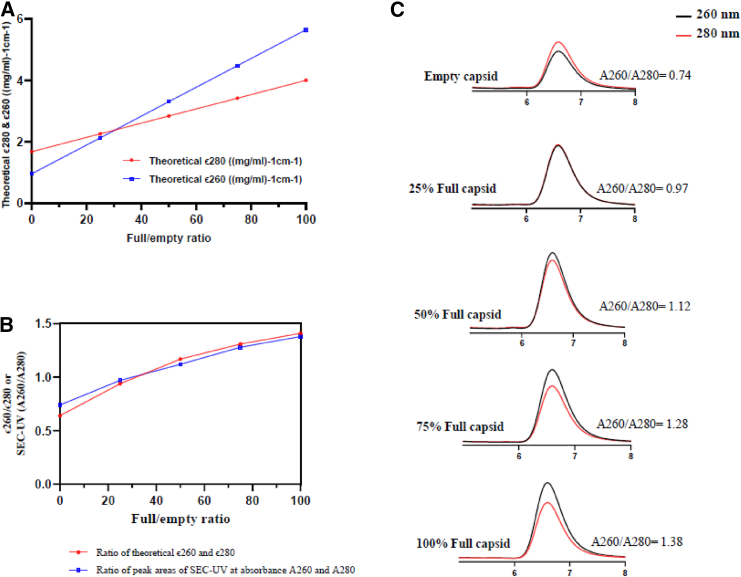
Correlation of full/empty ratio of rAAV with ECs (ϵ260, ϵ280) and absorbance peak areas of SEC-UV-HPLCs (at A260 nm, A280 nm) (A) Investigation of the relationship between different full/empty ratios of capsids and their corresponding theoretical ECs (ϵ) at 260 and 280 nm. (B) Correlation plot illustrating the relationship between the full/empty ratio of rAAV and the ratios of theoretical ECs (ϵ260/ϵ280) as well as the ratios of absorbance peak areas in SEC-UV-HPLC (A260/A280). (C) Chromatograms at SEC-HPLC-UV: comparison of rAAV empty (0%), full capsids (100%), and different levels of % full (25%, 50%, and 75%). The ratios of SEC-UV (A260/A280) shown in the figure were calculated using the peak areas obtained from SEC-UV analysis.

The full to empty capsid ratio of rAAV is an important attribute that has implications for product quality and safety.^20^^,^^21^ Therefore, establishing a method that can measure the full to empty capsid ratio with high accuracy, minimum sample preparation requirements, and standard analytical instrumentation is essential. Figure 5A shows the theoretical extinction coefficient at ϵ260, ϵ280 ((mg/mL)^−1^cm^−1^ plotted against the full AAV capsid percentage. The theoretical extinction coefficient as a function of % full capsid shows that the ϵ280 and ϵ260 plotted lines intersect at approximately 30% full capsid, indicating that at ∼30% of AAV full capsid the ϵ280 extinction coefficient) is equivalent to the ϵ260 extinction coefficient. The experimental ϵ280 of empty and full AAV capsids was determined using SEC coupled to UV, MALS, and RI detectors. The experimental (SEC-RI-MALS) derived ϵ280 of empty and full AAV capsids is 1.78 and 3.88, respectively. These experimental values are comparable to the theoretical ϵ280 of empty and full AAV capsid (1.68 and 4.0; see Figure 5A). The % difference between theoretical and experimental ϵ280 of empty and full AAV capsids is below 6% and 3%, respectively.

The SEC-UV (A260/A280) and theoretical ECs (ϵ260/ϵ280) ratio plots against % full AAV capsid are shown in Figure 5B. The two ratio plots in Figure 5B showed similar curve fittings red and blue traces, which demonstrates a good correlation between full and empty capsid ratio with AAV ECs ratios (ϵ260/ϵ280) and SEC-UV peak area ratio (A260/A280).

In addition, the SEC-UV (A260 and A280) chromatograms of empty (0%), spiked (25%, 50%, 75%), and full AAV (100%) are shown in Figure 5C. As shown in the chromatograms, the empty capsid has a higher SEC-UV absorbance peak at 280 nm (red trace) and lower SEC-UV absorbance peak at 260 nm (black trace). On the other hand, full AAV (100%) capsid has a higher SEC-UV absorbance peak at 260 nm (black trace) and lower SEC-UV absorbance peak at 280 nm (red trace). Interestingly, at ∼25% spike level of AAV capsid, the SEC-UV chromatographic traces at 260 nm (black trace) and 280 nm (red trace) are almost aligned on each other. This result is consistent with the previous observation in Figure 5A, which showed that, at ∼30% of AAV full capsid, the theoretical ϵ280 is equivalent to the theoretical ϵ260.

### Comparison of full and empty capsid ratio measurement of rAAV by SEC-UV (A260, A280)-MALS and other orthogonal techniques (qPCR/ELISA, AEX-HPLC)

**Figure 6:**
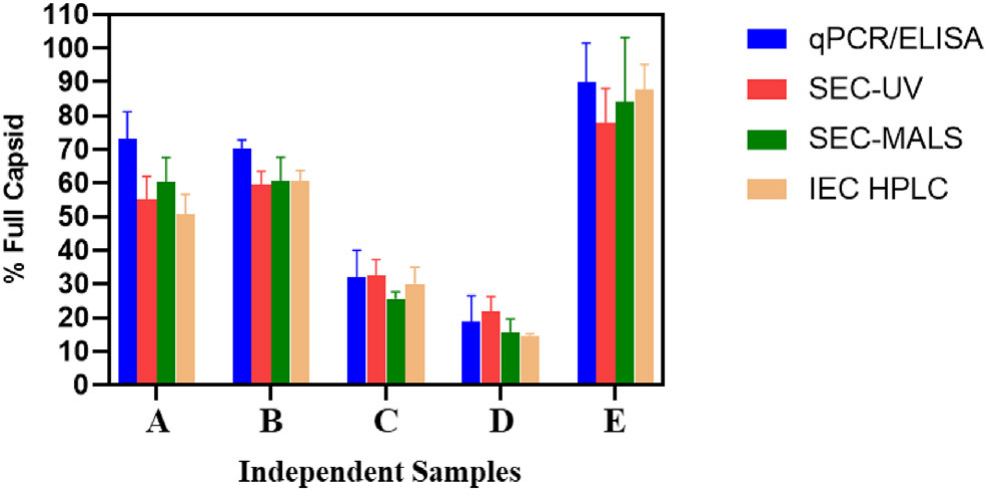
Comparative analysis of full/empty capsid ratio of rAAV Using qPCR/ELISA, AEX-HPLC, SEC-UV (A260/A280), and SEC-MALS methods

A set of five independent rAAV samples from different batches were used to evaluate the comparison of the F/E ratio. The percentage full capsid of the five samples was determined by SEC-UV peak area ratios (A260/A280), SEC-MALS, qPCR/ELISA ratio, and AEX-HPLC and are presented in Figure 6. For the SEC-UV (A260/A280) sample analysis, the F/E ratio was estimated using Figure 5B as a calibration curve. For the SEC-MALS, the F/E ratio was determined using the absolute molar mass obtained from the analysis. Overall, the results obtained by SEC-UV and SEC-MALS correlated well with the results from qPCR/ELISA and AEX-HPLC methods across the range of percentage full capsid. However, for sample E in Figure 6, we observed a lower F/E ratio by the SEC-UV method, which implies the method is better for the F/E range 0%–80% full capsid. For AAV samples with 80%–100% full capsid, the SEC-UV method with change in peak area ratio from A260/A280 to A260/A230 might give a more reliable quantification for F/E. Recently, published literature^22^ showed detailed studies to prove the hypothesis that SEC with UV (A260/A230) detector is better for quantifying higher full/empty ratio samples compared to A260/A280. The SEC-UV (A260/A230)-based calculations (for full/empty of AAV) have been published previously, so it was not evaluated again here.

### Monitoring and characterization of rAAV particles in downstream processing steps by SEC-UV-FLD method

**Figure 7:**
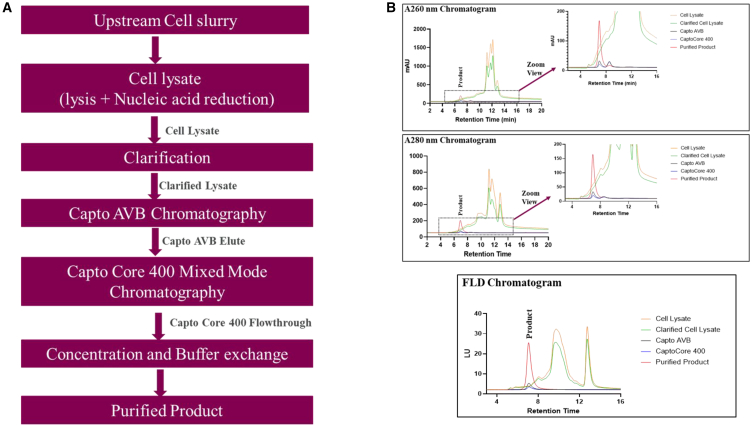
Downstream process overview and in-process monitoring by SEC-UV-FLD (A) An overview of the downstream processing steps involved in rAAV viral vector manufacturing. (B) SEC-HPLC (A260 nm, A280, and FLD) results for each intermediate step in the process (cell lysate, clarified lysate, affinity eluate, mixed-mode flow through, and purified AAV product).

A schematic overview showing a generic downstream process (DSP) of rAAV viral vector manufacturing is illustrated in Figure 7A. The analytical SEC coupled with UV (at 260 and 280 nm) and fluorescence detectors was used to evaluate the in-process samples generated at different DSP steps. The chromatographic profiles of SEC-UV-FLD of five in-process samples (cell lysate, clarified lysate, affinity resin elute, mixed-mode chromatography flow through, and purified rAAV product) are shown in Figure 7B. The profiles provide a snapshot of in-process samples and a rough estimate of virus content, as well as process-related impurities present (e.g., DNA, host cell protein [HCP], chromatin). Figure 7B also shows high abundant impurity peaks (from RT: 8–16 min) in cell lysate and clarified lysate samples compared to other downstream in-process samples. However, in the purified rAAV product sample, a single monomer peak (red trace in Figure 7B) was observed with a 98% purity level, demonstrating that the DSP steps were highly effective in removing and generating a highly purified rAAV product.

### Thermal stability and genome ejection of rAAV

AAV under thermal stress can eject viral genome from intact or partially disrupted AAV capsids. The thermal stability studies evaluated here can measure the initial and complete DNA ejection at various temperatures. The chromatograms from SEC-UV (A280) and SEC-UV (A260) help us to guide the DNA ejection temperatures and to determine the capsid stability.

**Figure 8:**
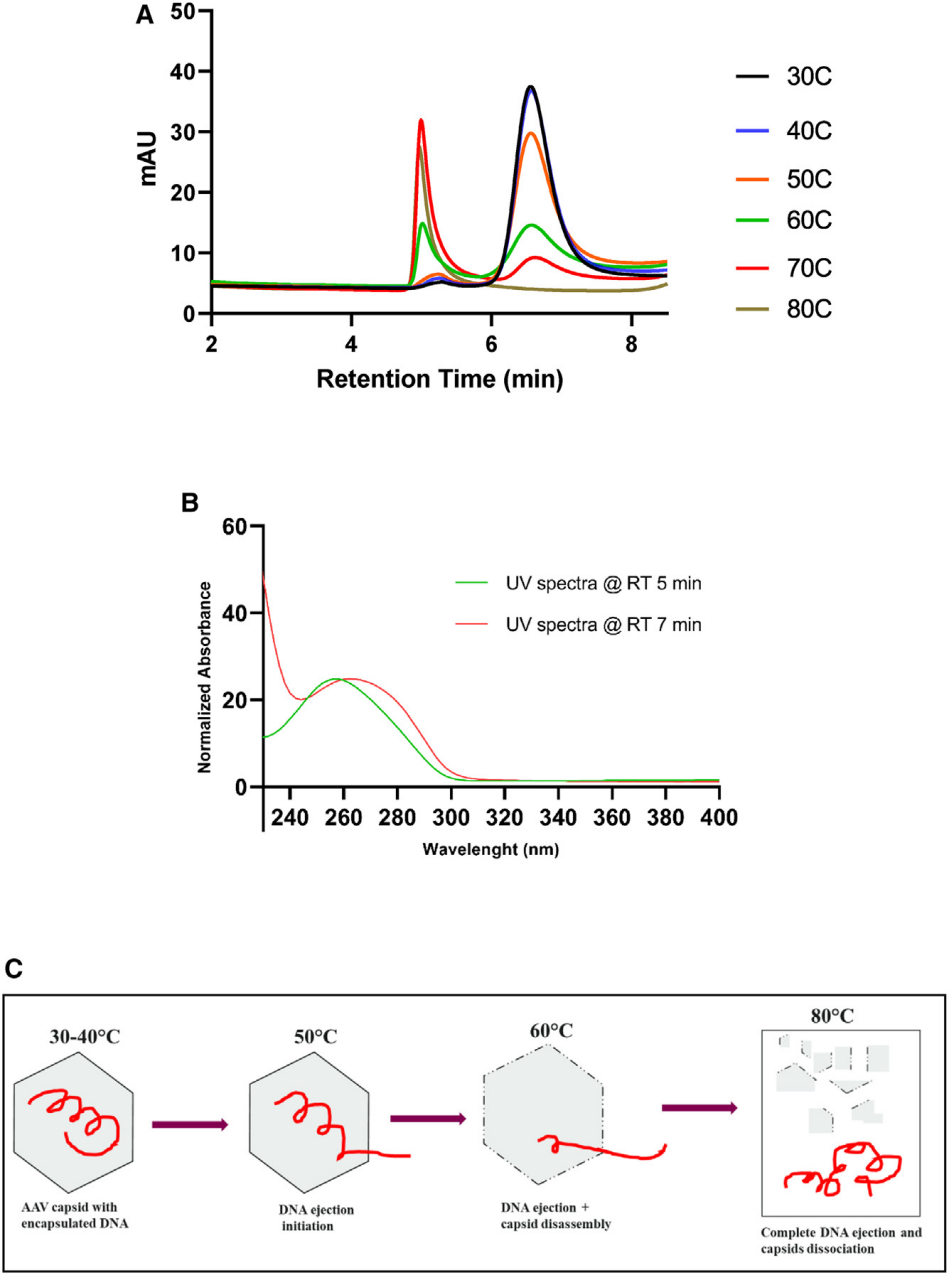
SEC-UV-HPLC analysis for measuring genome ejection and thermal stability of rAAV (A) SEC-UV (280 nm) chromatograms of rAAV samples incubated at various temperatures (30°C, 40°C, 50°C, 60°C, 70°C, and 80°C) for 1 h. (B) UV spectra of SEC chromatographic peaks for 80°C and 30°C samples, respectively. Green trace shows UV spectra with maximum absorbance at SEC-UV (A260 nm) (nucleic acid peak) at RT 5 min for 80°C sample and red trace shows UV spectra with maximum absorbance in range 270–280 nm (DNA + protein peaks) at RT 7 min for 30°C sample. (C) Visual representation of rAAV genome ejection under thermal stress, incorporating the findings from (A) and (B). As the temperature rises, beginning at around 40°C, the genome (ssDNA) is progressively ejected in a linear fashion. At approximately 80°C, complete genome ejection is observed, coinciding with the dissociation of the capsid. The dashed line on the capsid structure indicates the disassembly of the capsid during this process.

SEC-UV(280 nm) chromatograms of rAAV samples incubated for 1 h at different temperatures (30°C, 40°C, 50°C, 60°C, 70°C, and 80°C) are shown in Figure 8A. We observed a decrease in the monomeric peak at RT 7.0 min and an increase in the early-eluting peak at RT 5.5 min. At 30°C–40°C, the SEC-UV (A260/A280) ratio of the monomer peak was 1.20 representing ∼60% full, and the ratio decreased with an increase in temperature from 40°C to 70°C. At 80°C, the monomeric peak disappeared completely, and an increase in early-eluting peak at RT 5.5 min was observed with an SEC-UV (A260/A280) ratio >1.8. The higher UV (A260/A280) ratio (>1.8) suggests that the early-eluting peak is a nucleic acid-related peak. Figure 8B shows the UV spectra of the nucleic acid peak (at RT 5.5 min; green trace) and the monomeric peak (at RT 7.0 min; red trace) for 80°C- and 30°C-incubated rAAV samples, respectively. The UV spectra (Figure 8B) of the early-eluting peak (at RT 5.5 min) showed maximum absorbance at ∼260 nm. The UV spectra can be used as an orthogonal confirmation to support the hypothesis that the early-eluting peak contains nucleic acids. Based on the chromatographic profiles in Figure 8A and the decrease in both the % monomeric peak area and SEC-UV ratio (Figure S3), we demonstrated that the initial DNA ejection from intact AAV capsid starts above 40°C, followed by complete DNA ejection (with capsid disassembly) at 70°C–80°C. The graphical representation of AAV genome ejection is shown in Figure 8C.

## Discussion

AAV-based viral vectors have great potential to change the paradigm of drug development and delivery, bringing innovative medicines to patients.^1^^,^^2^ Due to the complex manufacturing process, achieving the production of consistent and high-quality AAV depends on the ability to rapidly characterize the PQAs of rAAV.^7^ Since AAVs have lower yields during production (cell culture and purification), an effective MAM method will be ideal to specifically monitor the PQAs of AAV during the process development, production, and storage. In that respect, an SEC-based separation method coupled in line with different detectors is well suited to monitor AAV PQAs with high throughput and low material demand, requiring only a single HPLC injection with adequate sensitivity.

SEC coupled in tandem with different detectors, such as UV, fluorescence, RI, and MALS, enables the measurement of multiple AAV product attributes, including purity (monomer, HMWs), apparent molar mass (Daltons), size in RMS radius of gyration (Rg), concentration (total capsid titer [vp/mL]; genome titer [vg/mL]), ECs (ϵ), and full and empty capsid ratio. In addition, this helps to enable rapid in-process monitoring (which provides confidence that unwanted components are removed during the DSP steps) and assessment of the thermal stability of AAV.

During the SEC method development, it was observed that improved chromatographic resolution was achieved with an isocratic mobile phase (phosphate buffer) with high salt concentrations for the separation of product-related impurities (HMWs from monomeric AAV). Usually, SEC resins with average pore sizes of 400–500 Å cannot separate proteins or protein-complexes with molecular weights of approximately 1.5–5 MDa. However, due to the compact AAV icosahedral shape (∼25 nm), the column pore size (400–500 Å) should be sufficient to separate AAV monomer, dimer, and oligomeric aggregates (∼100 nm).^23^ As aggregates size exceeds 100 nm, the SEC condition might disturb the large aggregates by trapping on the frits or packed bed of the column.^19^ Therefore, for larger aggregates (>100 nm), orthogonal assays such as field flow fractionation (FFF)-MALS should be used.^24^

In previous literature,^11^ the efficacy of the SEC method in distinguishing between monomeric, dimeric, and oligomeric aggregate species of rAAV has been highlighted. Our method has also been emphasized for its capability to effectively resolve different molecular forms of rAAV, aligning with the findings that spiking experiments with pure and aggregated rAAV materials result in good resolution and quantification of these species. This comparison with previous literature emphasizes the reproducibility and reliability of our SEC-HPLC method in rAAV analysis. Good linearity (R2 > 0.99) and recovery (90%–110%) were also observed for rAAV monomer and HMWs with this optimized SEC-HPLC method. These parallels further validate the utility of the SEC-HPLC method as a routine analytical tool for monitoring purity and HMW (<100 nm) levels in purified drug substances and drug products. The optimized method showed similar performance for different AAV serotypes, including AAV8 and AAV9 samples (Figure S4).

Other PQAs for AAV include absolute molar mass and RMS radius, which were measured by SEC-MALS without the requirement of any standards and were independent of column retention volume. Main monomer peak molecular weight (Mw) and RMS was measured as 4.2 MDa and 12 nm, respectively. The theoretical average molecular weight of AAV (∼50% full) is ∼4.3 MDa, confirming that the SEC-MALS analysis is capable of providing the expected absolute molar mass. Likewise, based on the theoretical rAAV size and previously published literature on orthogonal methods (MADLS/DLS),^11^^,^^25^ the results align well with the expected radius for AAV. The flat Mw and RMS profiles shows the main monomer peak as monodisperse, while the early-eluting HMWs peaks were identified as dimer, trimer, or oligomeric aggregates of AAV. The SEC-MALS confirmed the separation of size variants of rAAV such as monomer and HMW (or aggregates) forms.

To measure the total capsid titer (vp/mL) and genome titer (vg/mL) of rAAV, we developed SEC-FLD and SEC-UV (A260) methods orthogonal to traditional methods. A disadvantage of traditional method such as ELISA and qPCR is that they are labor intensive and have significant assay variability. In comparison with capsid ELISA and qPCR, the optical density (OD) method demonstrates less variability when measuring the concentration of AAVs.^10^ However, the OD method has a limitation when the AAV samples contain high levels of protein and nucleic acid impurities.^26^^,^^27^ Therefore, the SEC method with FLD- and UV (A260)-based detection have additional advantages over the OD method for the separation of capsid from impurities and excipients on the column. The linearity range of the standard curves for SEC-FLD and SEC-UV (A260) was established as 3.11 × 10^11^ to 7.5 × 10^13^ vp/mL and 8 × 10^10^ to 1.8 × 10^13^ vg/mL, respectively. Both standard curves have good linearity (R^2^ > 0.99) and precision (% RSD <10%).

For the extinction coefficient (ϵ) of AAVs, a detailed description is provided in the results section where the theoretical ϵ of empty and full AAVs were calculated at 260- and 280-nm wavelengths using previously published literature.^10^ Interestingly, when we used these theoretical ϵ and plotted them against the full and empty capsid AAV ratios, we observed that ϵ280 and ϵ260 lines intersect when 30% of capsids are full. The observations were further validated by spiking studies that showed that, at approximately 30% full rAAV, the SEC-UV chromatographic traces at 260 nm (black trace) and 280 nm (red trace) are almost indistinguishable. In addition, the present work also showed similar curve-fitting data, which demonstrate a good correlation between the ratio of theoretical ϵ260/ϵ280, the ratio of experimental peak areas of SEC-UV(A260/A280), and full/empty ratio of AAVs. To the best of our knowledge, this is the first study where the correlation was shown between theoretical ECs, ratio of experimental peak areas of SEC-UV, and full/empty ratio of AAVs.

Previous studies have established that A260/A280 ratios can indicate the filling status of AAV capsids, with lower ratios (∼0.59–0.7) associated with empty capsids and higher ratios (∼1.39–1.4) with full capsids. These findings are consistent with the A260/A280 ratios determined by AUC, which are 0.59 for empty and 1.39 for full capsids, demonstrating excellent agreement with the present work’s SEC-UV analysis.^16^^,^^28^^,^^29^ This consistency across different analytical methods highlights the reliability of the A260/A280 ratio as a marker for capsid content and purity.

To confirm the F/E capsid ratio of different rAAV batches, five different samples were tested with SEC-UV(A260/A280)-MALS. The results were compared with orthogonal methods including AEX-HPLC and qPCR/ELISA. The results from our studies showed a similar F/E ratio as compared with the AEX-HPLC and qPCR/ELISA results. For the qPCR/ELISA method, a slightly greater F/E ratio was observed. The increase in F/E ratio might be due to the combined assay variabilities from two separate measurements required to calculate the F/E ratio. Based on our previous publication^12^ and present AEX-HPLC results from the new sample sets, we observed baseline separation of empty and full rAAV species. In contrast, the SEC-HPLC has limitations in the separation of empty, partial, and full rAAV capsids. Despite this limitation, the F/E ratio could be accurately determined by utilizing SEC-UV (A260/A280) ratio. Moreover, the ability to utilize multiple detectors in tandem enabled greater throughput in the measurement of multiple attributes when compared to AEX-HPLC. Another added advantage is that the SEC-UV (A260/A280) and SEC-MALS methods are serotype independent, and as a result can be used to quantify the F/E ratios for any AAV serotypes (ranging from AAV1 to AAV9), requiring only minimal method optimization.

In this study, the potential of using SEC (A260/A280) ratios to differentiate between fully and partially filled AAV capsids was not investigated. However, in future, it will be worth exploring the direct correlation between A260 absorbance and the DNA content within the capsid, which influences the accurate quantification of partially filled capsids and their impact on full/empty (F/E) ratio assessments via SEC. Considering these limitations, alternative analytical approaches like mass photometry, analytical ultracentrifugation (AUC), and charge detection mass spectrometry (CDMS) should be considered for their enhanced accuracy in quantifying partial capsid populations.^15^^,^^30^^,^^31^^,^^32^

The SEC-UV(A260/A280)-FLD method described here also is suitable for analyzing in-process samples (in DSP) as a means to monitor virus content as well as impurity profiles (e.g., DNA, HCP, chromatin). In comparison to ELISA and qPCR, the SEC-HPLC method for in-process samples helps to provide a holistic view of components present in the in-process samples. The method is simple to perform, requires only minimal sample preparation, has a short analysis time, and is cost-effective. Previous studies^13^ also showed the superiority of HPLC-based methods, including anion, cation, and SEC, for monitoring in-process samples during the production and validation process of rAAVs. In the present study, the results obtained from in-process samples provide confidence that unwanted components are removed during the DSP steps starting from cell lysate to the final purified drug substance.

The purified rAAV product showed a single monomer peak with 98% purity. It is worthwhile to further explore the application of SEC-UV-MALS-FLD as a process analytical technology (PAT) tool during the production of rAAV. The SEC-HPLC-based PAT initiatives has significant advantages to provide real-time information that helps to better understand the process and ensure product quality output of the final drug substance (DS).

The AAV SEC analysis is experiencing rapid advancements by the development of novel column designs that incorporate reduced dimensions and stable, efficient particle materials. These improvements have greatly enhanced both the speed and resolution of AAV separations, with some studies reporting separation times below 10 min.^23^^,^^33^ However, when developing high-throughput methods to achieve separation times under 10 min, it is crucial to consider and optimize the method accordingly to address the unique challenges associated with AAV sample integrity. These challenges include non-ideal interactions with the column, elevated backpressure, and sensitivity to mechanical shear stress, all of which can adversely affect the quality and reliability of the analysis. Our present method employs a longer separation time of 30 min, although the majority of AAV separations occur within the first 6–8 min. The extended 30-min duration is intentional, as this method is designed to function as an MAM even for in-process downstream samples. The prolonged time frame facilitates the separation of process-related impurities, such as host-cell DNA and host-cell proteins, which elute up to 20 min, as illustrated in Figure 7B.

Another important factor to be monitored during the production and storage is the thermal stability of AAV products. The thermal stability of AAV has been previously examined by using differential scanning calorimetry (DSC), differential scanning fluorimetry (DSF), EM, atomic force microscopy (AFM), dynamic light scattering (DLS), SEC-MALS, nanoparticle tracking analysis (NTA), isothermal titration calorimetry (ITC), and infectivity technologies. With the use of DSF, DSC, and AFM, the melting temperature for AAV (different serotypes) was determined to be in the range from 65°C to 90°C.^34^^,^^35^^,^^36^^,^^37^^,^^38^ In this work, we go beyond these measurements by using SEC-UV (A280) chromatograms to monitor the disassembly of rAAV vector particles at increasing temperatures. The current study shows that the initial DNA ejection from intact rAAV capsid starts above 40°C followed by complete DNA ejection (with capsid disassembly) at 80°C. Our results align well with the previous literature,^39^ where a fluorescence-based assay (SYBR) showed that, at pH 7.0, DNA release from AAV started at 40°C and that complete DNA ejection was observed at 70°C–80°C. Therefore, the SEC-UV (A280) method can be used as a high-throughput method along with other biophysical techniques to predict the stability of rAAV during early-stage formulation screening and development.

In summary, the present work showed the capability of using the SEC-HPLC (coupled in line with multiple different detectors in sequence) method as an MAM to monitor different AAV PQAs throughout the AAV manufacturing process. The method showed similar results when compared with traditional, orthogonal methods for characterizing different attributes of AAV. The MAM-based workflow developed here is a valuable analytical tool for extended characterization of rAAV that is simple, label free, non-destructive, cost-effective, and requires limited amounts of sample. In addition, the method is a high-throughput method that can be applicable for all the serotypes of AAV in both process development and QC environment. Thus, the method can be used as a reliable routine method to support and guide the process and product development, formulation development, release, and stability studies during the production of AAVs.

## Materials and methods

### Chemical and reagents

HPLC-grade water (OmniSolv), sodium chloride, potassium chloride, sodium phosphate monobasic, and sodium phosphate dibasic were purchased from Sigma-Aldrich (Milwaukee, WI, USA). All other reagents used in the experiments were analytical grade from Sigma-Aldrich, JK Baker, or Alfa Aesar. Custom-made empty and full AAV6 construct were purchased from VIROVEK (Hayward, CA, USA) and formulated in PBS. The concentration of the full capsids was measured by the manufacturer as 2 × 10^13^ vg/mL.

### In-house rAAV production and purification

The rAAV vector was generated in house using the triple-plasmid transfection method.^5^ The resultant rAAV (AAV6.2) produced in house is a mutated-capsid variant (phenyalanine [F] residue at position 129 is substituted with leucine [L]) of wild-type AAV6 containing an encapsulated therapeutic transgene sequence.^20^ The rAAV vectors were produced using a pre-defined mixture of the three plasmids (inverted terminal repeat [ITR] vector with the gene of interest, AAV rep/cap, and Ad helper plasmid) in HEK293 cells, and the rAAVs were harvested at 48–72 h post transfection. After the harvest, the cell paste was stored at −80°C. To purify rAAV, cell paste was thawed, diluted to 10% of solid cell slurry, and then incubated with 0.5% polysorbate 80 and Benzonase at room temperature for 1 h. Before being loaded onto an affinity column, cell lysate was clarified with depth filter. Once the rAAV was eluted from the affinity column with 0.5 M NaCl at low pH, the rAAV product was further purified by mixed-mode chromatography, followed by concentration/buffer exchange in the formulation buffer. The final in-house rAAV DS was used for method development/qualification of the method.

Similarly, the highly aggregated rAAV material was generated using the same upstream and downstream processing steps as mentioned before. However, there are differences in the buffer-exchange step and formulation-buffer composition. The low concentration of PS-80 in the formulation buffer leads to the generation of highly aggregated rAAV.

### SEC-HPLC chromatographic method

#### Initial column screening and optimized method

An in-house rAAV reference material (manufactured using the above process) was used initially for the SEC-HPLC method development. Different columns with large pore size were used for the method development, including Sepax SRT SEC-500 (5 μm, 500 Å), SRT SEC-1000 (5 μm, 1,000 Å, 7.8 × 300 mm and 2.1 × 300 mm), TSKgel G5000PWxl (10 μm, 30 cm × 7.8 mm), Waters XBridge BEH SEC (450 Å, 3.5 μm, 7.8 × 300 mm), and ACQUITY UPLC Protein BEH SEC Column (450 Å, 2.5 μm, 4.6 × 300 mm). Different buffers (Tris, phosphate-buffered saline), flow rates, pH (6–7.5), ionic strength (KCl, NaCl), divalent cations (CaCl_2_, MgCl_2_), and other additives (e.g., arginine, acetonitrile) were evaluated to achieve the optimized condition for the separation of monomeric rAAV from aggregates (HMWs). During the SEC method development, it was observed that improved chromatographic resolution was achieved with an isocratic mobile phase (phosphate buffer) with high salt concentrations.

The final optimized SEC method was employed on a Sepax SRT SEC-500, 5 μm, 500 Å, (7.8 × 300 mm) column to separate AAV monomers and aggregates. The column was run on an isocratic mobile phase (20 mM sodium phosphate, 300 mM NaCl, 10 mM KCl, pH 7.0) at a flow rate of 1.0 mL/min for 30 min. The analysis was performed using an Agilent 1260 HPLC system with UV, fluorescence, and MALS detectors. More details regarding the detectors in combination with SEC-HPLC are provided in the MAM assay design section for extended characterization of rAAV PQAs.

### Viral genome titer (vg/mL) determination by qPCR

Samples were treated with DNase I (New England Biolabs) to remove free DNA and with Proteinase K (Invitrogen) to break down AAV capsids. Viral DNA was amplified using qPCR with a set of primers and TaqMan probe (5′ 6-FAM/3′ BHQ-1, Sigma-Aldrich) targeting the AAV-2 ITRs (sequences as in Limberis et al.^20^) using the QuantStudio 7 PCR System (Applied Biosystems). Purified linearized transgene plasmid was used as a standard to interpolate sample viral genome titers.

### Viral capsid titer (vp/mL) determination by ELISA

The Gyrolab Bioaffy sandwich immunoassay was performed on the Gyrolab xP workstation (Gyros, Uppsala, Sweden). Biotin anti-AAVX conjugate (Thermo Scientific) and ADK6 (Progen), an anti-AAV6-specific antibody conjugated in house to Alexa Fluor 647 (Invitrogen), were used as capture and detection reagents, respectively, together with Bioaffy 1000 CDs (Gyros AB). Biotin anti-AAVX conjugated was diluted to 100 μg/mL in the PBS/0.01% Tween buffer, while ant-AAV6-Alexa Fluor 647 antibody was diluted to 10 μg/mL in Rexxip F buffer (Gyros). The method setup followed a 3-step (capture-analyte-detect) run with two wash solutions used for washes after (capture-analyte-detect) following manufacturer’s instructions.

### MAM method assay design

#### Purity measurement (SEC with fluorescence detector)

The rAAV batch containing 45% of aggregation (or HMWs) was used for the initial development work. The higher aggregate level of the sample was confirmed using dynamic light scattering (DLS), AUC, and FFF-MALS orthogonal techniques before performing the SEC-HPLC experiments. For recovery (accuracy) experiments, a spiking study was conducted using an in-house rAAV purified reference (with 0% HMWs) and aggregated rAAV material (with 45% HMWs). The aggregated in-house rAAV material was spiked with 25% (v/v) and 50% (v/v) of pure rAAV reference material. Triplicate analyses were conducted for four samples: the rAAV purified reference (without spiking), 25% spike level, 50% spike level, and aggregated rAAV material, to assess the accuracy of the optimized SEC-HPLC method.

SEC column using fluorescence detection for rAAV offered significant improvements in sensitivity and specificity over UV detection by targeting the intrinsic fluorescence of capsid proteins. This technique minimizes interference from DNA content variability and UV-absorbing impurities, resulting in consistent and accurate quantification of rAAV titer and purity, even within complex matrices. Unlike UV detection, fluorescence provides a stable, DNA-independent signal, which is essential for reliable particle detection. Figure S5 demonstrates the capability of fluorescence detection to clearly distinguish rAAV particles in mixed-sample matrices. Overall, fluorescence-based SEC-HPLC represents a robust and precise alternative for rAAV purity assessment.

#### SEC-MALS analysis

For SEC-MALS analysis, aggregated rAAV material was used for absolute molar mass and RMS or Rg determination. An Agilent HPLC system was connected sequentially to multiple detectors including a UV detector and a Wyatt MALS Dawn EOS (18-angle static light scattering detector with built-in QELS DLS detector and RI detector). The output signals from the UV, MALS, and RI detectors were imported into the ASTRA 6.1 software. The MALS and RI signals were normalized during the data processing to determine the average absolute molar mass and RMS radius of each peak separated on the SEC column. In addition to molar mass and RMS radius, the monodisperse (i.e., single population) and polydisperse (i.e., mixed populations) properties of each peak were also profiled during the SEC-MALS analysis to give greater insight into the exact composition of the sample. The light-scattering detectors were normalized using bovine serum albumin (2 mg/mL) before the sample analysis.

For extinction coefficient (ϵ) determination experiments, the dn/dc (specific RI increment) values of 0.185 (empty AAV) and 0.170 (pure nucleic acid) were applied. Both dn/dc values were based on reported literature values.^27^ In addition, a “sphere” model was used with the ASTRA 6.1 software to calculate the experimental ϵ for icosahedral AAV.

#### Linearity and titer measurements for in-house rAAV

An in-house rAAV purified reference was used for the preparation of calibration curves (7 serial dilution points) and determination of the performance parameters of the method. The initial total capsid titer (by ELISA) and viral genome titer (by qPCR) of rAAV reference stock material were pre-determined as 7.5 × 10^13^ vp/mL and 1.83 × 10^13^ vg/mL, respectively. Three independent calibration curves were prepared separately using a serial dilution of in-house rAAV reference stock material ranging from 0.29 × 10^12^ to 7.50 × 10^13^ vp/mL (or 0.71 × 10^11^ to 1.83 × 10^13^ vg/mL) using formulation buffer as a dilutant. The individual calibrated points (or serially diluted samples) were loaded onto the SEC column, and the elution peaks were monitored with fluorescence detection for total capsid titer vp/mL and UV-A260 detection for viral genome titer vg/mL.

For the comparison of the concentration or titer measured by traditional methods with the newly developed SEC-UV-FLD method, we used four independent in-house rAAV samples. The samples were tested side by side with ELISA, qPCR, and SEC-UV (A260)-FLD. Each sample was analyzed in triplicate in each of the assays, and the average titer data (vp/mL, vg/mL) was plotted for comparison.

#### ECs (at absorbance A260, A280) and full/empty ratio of AAV

AAV vectors are a combination of capsid proteins and encapsulated DNA. The extinction coefficient (ϵ) for AAV was calculated using the combined extinction coefficient of the protein-DNA complex.^10^ For empty AAV capsids, which consist mostly of capsid proteins, the theoretical extinction coefficient at 280 nm (ϵ_280_) was determined based on the absorptivity of the aromatic amino acids present in the primary sequences of VP1, VP2, and VP3 (assuming a VP1:VP2:VP3 ratio of 1:1:10). The extinction coefficient at 260 nm (ϵ_260_) of empty AAV capsid was calculated based on the extinction coefficient at 280 nm (ϵ_280_) and a previously published conversion factor of 0.59.^10^ For full AAV capsids (containing protein and encapsulated DNA), the theoretical ECs ϵ280 and ϵ260 were calculated using published literature according to the following formula:

(Equation 1) $$\epsilon 260\;\left(\text{DNA}\;\text{containing}\;\text{AAV}\right)=20.0\times {\text{MW}}_{\text{DNA}}+3.72\times 10^{6}\times \text{vp}/\text{vg}$$

(Equation 2) $$\epsilon 280\;\left(\text{DNA}\;\text{containing}\;\text{AAV}\right)=11.1\times {\text{MW}}_{\text{DNA}}+6.31\times 10^{6}\times \text{vp}/\text{vg}$$

where MW_DNA_ is molecular weight of the vector genome.

Once the molar extinction coefficient (units: M^−1^cm^−1^) of AAV was calculated using Equations 1 and 2, the molar extinction coefficient was converted into the calculated theoretical extinction coefficient (units: (mg/mL)^−1^cm^−1^) by dividing the molar extinction coefficient with the molecular weight (Da) of full capsids. Based on the above considerations and conversion factor for empty capsids, the calculated theoretical extinction coefficient for empty capsid is determined as 1.68 (ϵ280) and 0.98 (mg/mL)^−1^cm^−1^ (ϵ260), respectively. For the AAV full capsid (100% encapsulated), the calculated theoretical extinction coefficient is determined as 4.0 (ϵ280) and 5.65 (mg/mL)^−1^cm^−1^ (ϵ260), respectively. The theoretical ECs ϵ260 and ϵ280 were plotted against the theoretical full/empty capsid ratio of AAV. The experimental ECs of AAV empty and full capsid were determined using SEC coupled in line to UV, MALS, and RI detectors.

#### Determination of full/empty ratio of rAAV by SEC-UV (A260, A280)

Based on Beer-Lambert’s law, the absorbance (A) at a specific wavelength depends on the molar extinction coefficient, concentration of the sample, and optical path length.^22^ Therefore, the peak areas observed on SEC-UV (A260) and SEC-UV (A280) will have a linear relationship with the ECs of the AAV samples (empty and full capsid).

To validate this hypothesis and demonstrate the applicability of the method, the empty (0%) and full capsids (∼100%) along with spiked rAAV samples (25%, 50%, 75% full) were analyzed by SEC-UV (A260) and SEC-UV (A280). Before performing the spiking experiments, the concentration of empty and full capsid of rAAV was determined using the in-house SEC-FLD method as described previously. Based on the SEC-FLD-determined concentration, the empty and full rAAV samples were diluted with PBS buffer to achieve a similar vp/mL concentration. The empty capsid of rAAV (0%) was spiked (vol/vol) with full AAV capsid (∼100%) to achieve approximately 25%, 50%, and 75% of full rAAV capsid. The experimental peak area ratios obtained from SEC-UV (A260/A280) were plotted with the theoretical extinction coefficient ratios at ϵ260/ϵ280 and full/empty ratio of AAV.

#### Quantification of the full/empty rAAV ratio by various orthogonal assays

The relative quantitation of the full and empty capsid ratio measured by the SEC-UV (A260/A280) method is compared with orthogonal methods such as the qPCR/ELISA titer ratio method and anion-exchange (AEX)-HPLC-based full and empty capsid ratio measurements. These two orthogonal methods are widely accepted methods for the quantification of empty and full capsids. Our lab previously published an AEX-HPLC method to measure the full and empty capsid ratio of rAAV. The paper presented extensive data that compared the full/empty ratio measurements by AEX-HPLC with the traditional AUC method, which is considered the gold standard for full/empty AAV capsid characterization. Therefore, for this study, we only used the AEX-HPLC method for comparison instead of the AUC method.^12^ A set of five independent in-house rAAV samples from different batches (with varying full/empty ratios) was used to evaluate the comparison of the full/empty ratios.

#### In-process monitoring of product (rAAV) in the DSP

For in-process sample testing, the samples were collected from each downstream processing step and analyzed with the SEC-UV-FLD method. Cell lysate and clarified lysate samples were centrifuged, and the supernatant was used for SEC-UV-FLD analysis. All other samples starting from the Capto AVB elute were injected neat onto the SEC column without any further dilutions. The overview of DSP steps is shown in the results section (Figure 7A).

#### Preparation of thermal stress samples

AAV vectors have previously been shown to undergo structural transition when incubated at higher temperatures.^24^^,^^25^ We adapted this approach to determine the thermal stability of AAV samples. An in-house purified rAAV reference was sub-aliquoted and incubated for 1 h at 30°C, 40°C, 50°C, 60°C, 70°C, and 80°C. The thermal stress samples were loaded onto the SEC column and the peaks were monitored with the SEC-UV(A260 and A280) detection.

## Data and code availability

All the data generated or analyzed for this study are available in the published article and its supplementary information files. For further inquiries or additional information, interested individuals can contact the corresponding author and submit a reasonable request.

## Acknowledgments

This research was developed with funding from the 10.13039/100000185Defense Advanced Research Projects Agency under HR011-18-3-001. The views, opinions, and/or findings expressed are those of the authors and should not be interpreted as representing the official views or policies of the Department of Defense or the US government.

## Author contributions

Conceptualization and methodology, S.H.R.M., A.P., and T.W.; writing – original draft, S.H.R.M.; writing – review & editing, S.H.R.M., A.P., T.W., N.B., X.C., T.L., A.S., and W.X.; resources, G.X. and T.L.; and supervision, X.C. and W.X.

## Declaration of interests

All authors are full-time employees or formal employees and may be shareholders of AstraZeneca.

## Supplemental information

Document S1. Figures S1–S5

Document S2. Article plus supplemental information
