# Supplementary material for: Size-exclusion chromatography as a multi-attribute method for process and product characterization of adeno-associated virus
Source: Mol Ther Methods Clin Dev. 2024 Nov 19;32(4):101382. doi: 10.1016/j.omtm.2024.101382 (PMC11647602; doi:10.1016/j.omtm.2024.101382)
Supplement: Document S1. Figures S1–S5 [file mmc1.pdf]

OMTM, Volume *32*

Supplemental information

Size-exclusion chromatography as a multi-attribute method for process and product characterization of adeno-associated virus

Sri Hari Raju Mulagapati, Arun Parupudi, Tomasz Witkos, Nick Bond, Xiaoyu Chen, Thomas Linke, Guoling Xi, Albert Ethan Schmelzer, and Wei Xu

<u>**Supplemental Material:**</u>

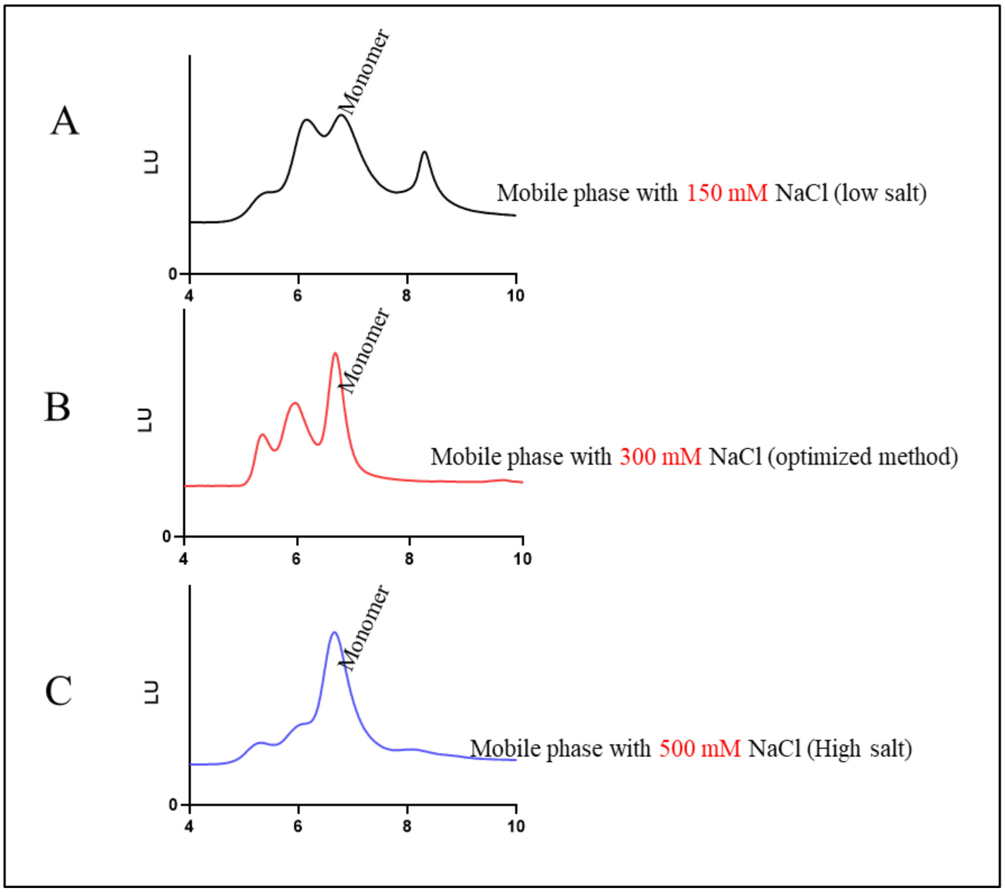

Figure S1: Comparative analysis of AAV6.2 separations using varying NaCl salt concentrations run on Sepax SRT SEC-500, 5µm, 500 Å (Optimized method) column

(A) Mobile phase with lower NaCl concentration (20 mM sodium phosphate, 150 mM NaCl, 10 mM KCl, pH 7.0)

(B) Optimized condition (20 mM sodium phosphate, 300 mM NaCl, 10 mM KCl, pH 7.0)

(C) Mobile phase with higher NaCl concentration (20 mM sodium phosphate, 500 mM NaCl, 10 mM KCl, pH 7.0)

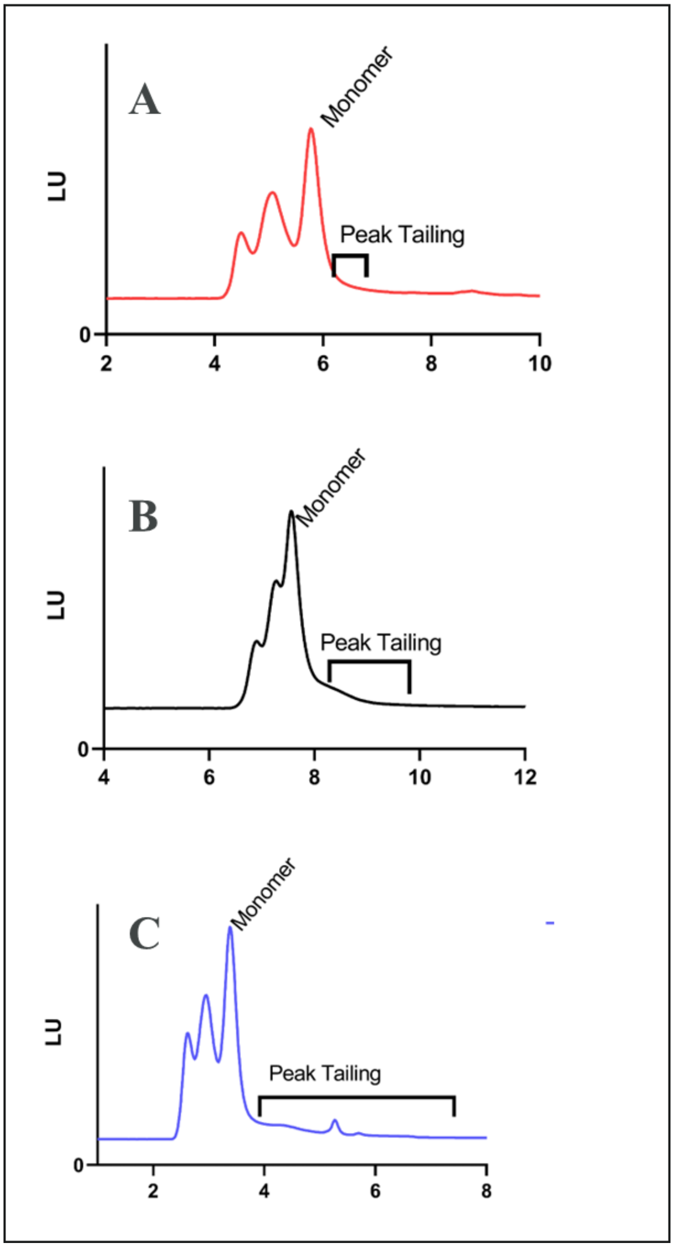

Figure S2: AAV6.2 separations using different columns (pore and particle size)

(A) Sepax SRT SEC-500, 5µm, 500 Å (Optimized method)

(B) Sepax SRT SEC-1000, 5µm, 1000 Å

(C) Waters XBridge BEH SEC (450 Å, 3.5 µm, 7.8 mm x 300 mm)

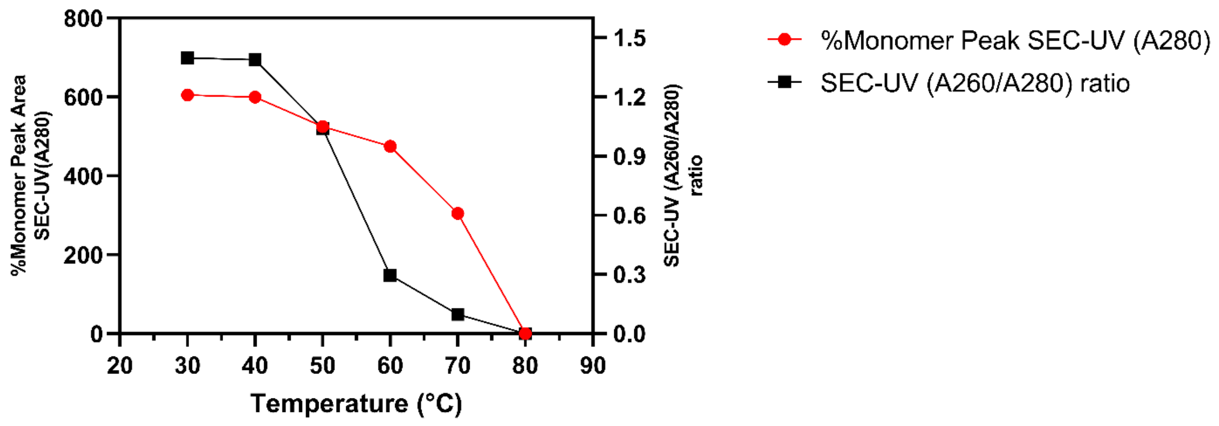

Figure S3: SEC-UV(A260 and A280)-HPLC for assessing the thermal stability of rAAV under elevated temperature stress. The figure depicts the relationship between the peak areas of SEC-UV(A280) and the peak area ratios of SEC-UV(A260/A280) at various elevated temperatures.

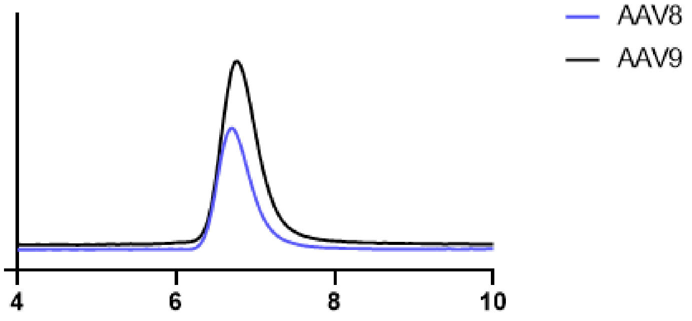

<u>Figure S4:</u> AAV SEC-FLD profiles for AAV8 and AAV9 serotypes

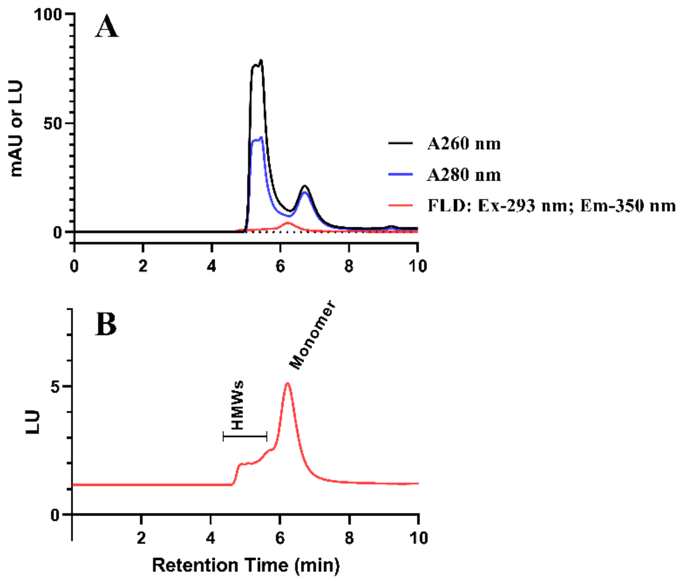

<u>Figure S5:</u> SEC coupled with UV (A260 and A280) and fluorescence detector for measuring rAAV monomer purity and HMWs level in the presence of sample matrix (Formulation buffer) with UV absorbing excipients/impurities

A) Overlay of SEC profiles displaying fluorescence emission spectrum (red) and UV spectra at 260 nm (black) and 280 nm (blue).

B) Fluorescence emission spectrum illustrating monomer purity (70%) and HMWs level (30%).
